# Minutes of the Fifth Annual Meeting of the Ohio State Dental Society, Columbus, O., Dec. 6, 1870

**Published:** 1871-03

**Authors:** W. M. Herriott


### MINUTES OF THE FIFTH ANNUAL MEETING OF THE OHIO STATE DENTAL SOCIETY, COLUMBUS, O., DEC. 6, 1870.

The Society met at 10 A. M., and was called to order by Dr. F. H. Rehwinkle, President. Roll being called, the following members were present: Drs. J. Taft, H. A. Smith, Geo. Watt, N. W. Williams, A. A. Blount, M. DeCamp, C. H. Harroun, F. H. Rehwinkle, B. T. Spellman, I. Williams, Will Taft, W. M. Herriott, D. R. Jennings, J. C. Whinnery, E. C. Sloan and H. Scott.

Minutes of the last meeting were read and approved.

Dr. Herriott, Chairman of Committee on Membership, requested all who desired membership in this society, to remain after adjournment.

Adjourned, to meet a 2 P. M.

### AFTERNOON SESSION, 2 O'CLOCK.

The meeting was called to order by President, Dr. Rehwinkle, and opened with prayer by the Rev. Mr. Spahr.

Minutes of morning session read and approved.

The President then delivered his annual address, which was full of good thoughts and suggestions, and was well received.

The Committee recommended the following persons for membership in this society: Drs. W. H. Sedgwick, Granville; L. M. Gray, Zanesville; R. A. Bonifield, Zanesville; F. S. Whitsler, Youngstown; J. M. Porter, Massillon, and A. F. Price, Fremont, all of whom were unanimously elected members.

On motion, Dr. J. H. Warner, of St. Clair, Michigan, was invited to be present as a corresponding member.

Dr. D. Phillips, of Springfield, was, upon recommendation, elected a member.

Dr. Buffett, introduced the following resolution:

*Resolved*, That no one shall hereafter become a member of this society, unless he has a diploma, from a reputable dental college, or a certificate from the State Board of Examiners.

Which was, after some discussion, upon motion of Dr. Keely, postponed until 2 o'clock, Wednesday.

Essays being called for, Dr. Watt read a paper, subject: "Thoughts on Respiration," which was listened to with great interest, and on motion, was referred to the Publishing Committee.

On motion of Dr. Harroun, the thanks of this society were presented to Dr. Watt, for the able and instructive manner in which he presented this very important subject. It was then discussed by Drs. Taft, Watt and Spellman.

Adjourned to meet at 7 o'clock.

EVENING SESSION, 7 O'CLOCK.

Society met as per adjournment; was called to order, and business commenced by reading the minutes of afternoon session, which were approved.

Committee on Membership reported the names of Drs. D. L. Pratt, Bethesda; S. H. Harlan, Mechanicsburg; A. Spencer, Columbus, who were unanimously elected members.

On motion of Dr. J. Taft, the reading of essays was postponed, and time fixed was Wednesday afternoon; and the first subject, "Diseases of the Antrum and Treatment," was then discussed by Drs. Buffett, Herriott, Butler, Jennings, Watt, J. Taft, DeCamp, Rehwinkle and Prof. Reamy, of Starling Medical College, who interested the society at length.

On motion adjourned, to meet at 8 A. M., to-morrow, Wednesday, for clinics.

WEDNESDAY, 2D DAY—8:45 A. M.

President Rehwinkle in the chair.

Society was called to order, when prayer was offered by Rev. Mr. Spahr.

Clinics being the order, occupied the time until 12 M.

Adjourned, to meet at 2 P. M.

AFTERNOON SESSION, 2 O'CLOCK.

Called to order by Dr. Buffett, 1st Vice-President.

Prayer by Rev. D. Kingery.

Committee on Membership reported the names of L. T. Beilharz, Fremont; G. A. Curtis, Gallion; M. A. Spencer, Orville; B. F. Rosson, Troy, all of whom by a unanimous vote were elected members.

On motion of Dr. J. Taft, Dr. Buffett's resolution defining membership, was taken up and passed.

The time for essays having arrived, Dr. D. R. Jennings presented an able paper, subject: "Dental Fees," which was received by the society, and discussed by Drs. Whinnery, I. Williams, Spellman, Sedgwick, Keeley, Blount and N. W. Williams, after which it was referred to the Publishing Committee.

Next was read a paper by Dr. Herriott, subject: "Artificial Dentures," which was received and discussed at length by Drs. Blount, Smith, Watt, Spellman, Bailey, Warner, Herriott and Horton, and was referred to the proper committee. The President reported the following committee to nominate members of State Board of Examiners: Dr. Geo. Keely, Oxford; Dr. L. Buffett, Cleveland; Dr. E C. Sloan, Ironton. Committee to nominate officers for State Society: Dr. Blount, Springfield; Dr. Harroun, Toledo; Dr. Watt, Cincinnati.

Adjourned, to meet at 7 P. M.

EVENING SESSION, 7 O'CLOCK.

President Rehwinkle in the chair.

Minutes of previous session read and approved.

Reading of essays being resumed, Dr. Butler presented an essay, subject: "Discipline of Head, Heart and Hand,"

which was received and discussed by Drs. Watt, Spellman, Butler, Sedgwick, Herriott, J. Taft, Smith, Keely, Horton and DeCamp, after which it was referred to the Publishing Committee.

Committee on Membership presented the names of the following, who were elected members: E. Chidister, Massillon; F. C. Fahnistock, Cincinnati; W. J. Moffitt, Coshocton; J. T. McCarroll, Bucyrus; S. B. Lewis, Greenville.

The fourth subject for discussion, "Anæsthetics in Dentistry and Minor Surgery," was then taken up and discussed by Drs. Watt, Keely and Rosson.

Adjourned, until 9 o'clock to-morrow, (Thursday) morning.

THURSDAY, 3D DAY—MORNING SESSION.

President Rehwinkle in the chair.

Rev. Mr. Spahr offered prayer.

Minutes of previous session read and approved.

W. A. Porter, of Dalton, was reported for membership, and elected.

Committee to nominate persons suitable to fill vacancies occurring in Board of State Examiners, reported, that in view of the fact that the present members of said board are working efficiently, and harmoniously, we would therefore recommend the re-election of the retiring members, Dr. M. DeCamp. and W. P. Horton.

GEO. W. KEELY,<br>L. BUFFETT,<br>E. C. SLOAN. } *Committee.*

Which was received, and a ballot being had, resulted in the election of Drs. DeCamp and Horton, to act as members of State Board of Examiners, for the term of three years.

Committee to nominate officers for coming year reported, and time for election fixed, 11 o'clock.

Drs. Keely, Porter, J. Taft, Blount, Whitsler, Horton, Harroun, C. R. Taft, Watt, Pierce, Butler, Whinnery, Sloan and H. A. Smith, were elected delegates to the American Dental Association.

The second subject: "Uses of Mallet in Excavating and Filling Teeth," was next discussed by Drs. Spellman, Moffitt, Whinnery, Lilly, Harroun, Kelsey, Rosson, Herriott, C. R. Taft, Blount, Butler, Rehwinkle and Horton.

The hour having arrived to elect officers for ensuing year, the following were elected:

*President*—Dr. Geo. W. Keely, Oxford.

*First Vice-President*—Dr. D. R. Jennings, Ravenna.

*Second Vice-President*—C. R. Taft, Mansfield.

*Recording Secretary*—W. M. Herriott, Zanesville.

*Treasurer*—Geo. Watt, Cincinnati.

On motion, Mr. Pack, of New York, was invited to address the society, in regard to "Eureka gold," when he spoke at length upon the subject of "gold foils." He was followed by Dr. Butler, of Cleveland, who gave his experience in operating with Mr. Pack's preparation of gold, which was favorable to it.

Dr. Buffett explained a mode of filling porcelain teeth.

Dr. Horton presented the following resolutions, which were passed:

*Resolved*, That the Committee on Publication is hereby authorized and requested, to furnish one hundred blank certificates, for use of the Examining Board; that said blanks be furnished before the 1st of June, 1871, and that where present blank reads, "A law entitled a law, etc.," be made read, "A law entitled an act, etc."

*Resolved*, That the Examining Board is hereby authorized to audit and pay its own expenses, from funds that may come into its hands from time to time; subject, in all cases, to the approval of the State Dental Society.

Adjourned, to meet at 2 P. M.

AFTERNOON SESSION, 2 O'CLOCK.

Society called to order by Vice-President, Buffett.

Bills were presented for hall rent, reporter and janitor, and were audited, and orders drawn to pay the same, amounting to sixty-five dollars, ($65).

Dr. Hollingsworth, of Indianapolis, was then called upon to explain his mode of mounting teeth upon aluminum plates, which he did to the edification of the members of the society.

On motion of Dr. Keely, the thanks of the society were tendered to Col. E. J. Blount, proprietor of the American House, for his kindness to, and his efforts for the comfort of the members during their stay with him.

Dr. J. Taft, President, and Dr. Horton, Secretary of the State Board of Examiners, made a report, giving names of those who have been examined, and have been granted certificates to practice dentistry, under the State law, which was as follows:

The Board of Examiners beg leave to report that the following persons have been examined, and being found qualified to practice dentistry, have received the certificate of the Board, according to the requirements of the law:

1868.

July 8. Thomas M. Talbott, Galion, Crawford Co.
Dec. 2. Joseph L. Dunlap, Chillicothe, Ross Co.
" " Israel Williams, New Philadelphia, Tuscarawas Co.
" " T. W. French, Chillicothe, Ross Co.
" " J. C. Hardy, Cincinnati, Hamilton Co.
" " John L. Scott, Defiance, Defiance Co.

1869.

Dec. 8. W. P. Hall, Piqua.

" " H. M. Reid, Cedarville, Greene Co.

" " J. McKinley, Uhricsville.

" " A. R. Lord, Shelby.

" " S. J. Way, Waynesville.

" " E. J. Way, Sandusky.

1870.

June 30. Owen Thomas, Yellow Springs, Green Co.
" " J. A. VanVleck, Gallipolis, Gallia Co.

June 30. Rob't H. Boal, Urbana, Champaign Co.
" " E. Chidester, Massillon, Stark Co.
Dec. 7. J. H. Siddall, Canton, Stark Co.
" " F. C. Fahnistock, Cincinnati, Hamilton Co.
" " J. S. McCarroll, Bucyrus, Crawford Co.
" " W. J. Moffitt, Coshocton, Coshocton Co.
" " M. A. Spencer, Orville, Wayne Co.
" " G. A. Curtis, Galion, Crawford Co.
" " M. Jefferson, Cambridge, Guernsey Co.
" " S. B. Lewis, Greenville, Darke Co.
" 8. W. F. Semple, Mt. Vernon, Knox Co.
" " Robt. W. Stephens, Mt. Vernon, Knox Co.

J. Taft, *Pres.*

W. P. Horton, *Sec., S. B. E.*

REPORT OF THE TREASURER OF THE BOARD.

Ohio State Dental Society in account with W. P. Horton, Treasurer of Examining Board.

| 1870. | | Dr. |
|---|---|---|
| May 18. | Express charges to Cincinnati | $ 25 |
| June 10. | Express charges to Cincinnati | 25 |
| " 28. | Paid Cleveland Leader Co. for printing circulars | 20 00 |
| " 30. | Paid J. Taft, according to resolution | 40 00 |
| " 30. | Paid W. P. Horton | 38 00 |
| " 30. | Paid M. DeCamp | 16 00 |
| " 30. | Paid F. H. Rehwinkle | 18 00 |
| " 30. | Paid H. A. Smith | 10 00 |
| | | 142 50 |
| Dec. 8. | Paid Treasurer State Society | 233 50 |
| | | $375 00 |

| 1870. | | Cr. |
|---|---|---|
| Dec. 8. | By cash balance | $ 25 00 |
| June 30. | By cash from examinations | 100 00 |
| Dec. 8. | By cash from examinations | 250 00 |
| | | $375 00 |

Respectfully submitted,

W. P. HORTON, *Treas. S. B. E.*

Which was ordered on file.

Dr. Geo. Watt, Treasurer, reported, which report being referred to Auditing Committee, was referred back to Treasurer after being found correct, and was as follows:

| | |
|---|---|
| Balance in treasury from 1869 | $ 3 12 |
| Dues paid in 1870 | 150 00 |
| From Treasurer State Board | 233 50 |
| Total | 386 62 |
| Bills paid | 382 05 |
| Balance | $4 57 |

A vote of thanks was tendered to Rev. Mr. Spahr.

The time having arrived for the officers elect to enter upon their official duties, Dr. Geo. W. Keely, President elect, was conducted to the chair by Drs. Spellman and Blount, where he was received by the retiring President, Dr. Rehwinkle, in a very warm-hearted and complimentary speech.

A vote of thanks was tendered to Dr. Rehwinkle for the able manner in which he had presided as President.

The President announced the following as standing committees for ensuing year:

*Executive Committee.*—W. M. Herriott, C. R. Taft and W. P. Horton.

*Membership.*—B. T. Spellman, L. Buffett, I. Williams.

*Dental Ethics.*—A. A. Blount, B. F. Rosson, C. Bradley.

*Publishing.*—J. Taft, Geo. Watt, N. W. Williams.

The following were appointed essayists for next annual meeting: H. A. Smith, C. R. Taft, A. A. Blount, Geo. Watt, W. P. Horton, L. Buffett, J. C. Whinnery.

Adjourned, at 5 P. M., to meet on the first Tuesday of December, 1871.

W. M. HERRIOTT, *Rec. Sec.*

